# Real-Life Outcomes of Coronary Bifurcation Stenting in Acute Myocardial Infarction (Zabrze–Opole Registry)

**DOI:** 10.3390/jcdd8110155

**Published:** 2021-11-11

**Authors:** Wojciech Milejski, Jerzy Sacha, Piotr Feusette, Marek Cisowski, Piotr Muzyk, Andrzej Tomasik, Marek Gierlotka, Beata Morawiec, Damian Kawecki

**Affiliations:** 1 2nd Department of Cardiology, Faculty of Medicine in Zabrze, Medical University of Silesia in Katowice, M. Skłodowskiej-Curie 10, 41-800 Zabrze, Poland; milejski_wojciech@o2.pl (W.M.); piotrekmuzyk1989@gmail.com (P.M.); tomasik@poczta.onet.pl (A.T.); beamorawiec@wp.pl (B.M.); 2 Department of Cardiology, University Hospital in Opole, W. Witosa 26, 45-401 Opole, Poland; j.sacha@po.edu.pl; 3 Faculty of Physical Education and Physiotherapy, Opole University of Technology, Prószkowska 76, 45-758 Opole, Poland; 4 Department of Cardiology, University Hospital, Institute of Medical Sciences, University of Opole, W. Witosa 26, 45-401 Opole, Poland; piotr.feusette@uni.opole.pl (P.F.); marek.gierlotka@gmail.com (M.G.); 5 Department of Cardiac Surgery, University Hospital, Institute of Medical Sciences, University of Opole, W. Witosa 26, 45-401 Opole, Poland; mcisowski58@gmail.com

**Keywords:** acute myocardial infarction, coronary bifurcation, percutaneous coronary intervention, target lesion failure

## Abstract

Percutaneous coronary intervention (PCI) of bifurcation lesions is a technical challenge associated with high risk of adverse events, especially in primary PCI. The aim of the study is to analyze long-term outcomes after PCI for coronary bifurcation in acute myocardial infarction (AMI). The outcome was defined as the rate of major adverse cardiac event related to target lesion failure (MACE-TLF) (death-TLF, nonfatal myocardial infarction-TLF and target lesion revascularization (TLR)) and the rate of stent thrombosis (ST). From 306 patients enrolled to the registry, 113 were diagnosed with AMI. In the long term, AMI was not a risk factor for MACE-TLF. The risk of MACE-TLF was dependent on the culprit lesion, especially in the right coronary artery (RCA) and side branch (SB) with a diameter >3 mm. When PCI was performed in the SB, the inflation pressure in SB remained the single risk factor of poor prognosis. The rate of cumulative ST driven by late ST in AMI was dependent on the inflation pressure in the main branch (MB). In conclusion, PCI of bifurcation culprit lesions should be performed carefully in case of RCA and large SB diameter and attention should be paid to high inflation pressure in the SB. On the contrary, the lower the inflation pressure in the MB, the higher the risk of ST.

## 1. Introduction

Although the optimal strategy for the treatment of coronary bifurcation is rather established, PCI of bifurcation as the culprit lesion in AMI remains a technical challenge [1], as it carries additional risk deriving from the specificity of acute conditions and the relatively little time for planning the procedure and for the restoration of the blood flow. Robust data on pathophysiology and advances in treatment, strategy and devices do not fully cover the population of AMI with bifurcation culprit lesion, as these patients are mostly underrepresented in opinion-making trials. Therefore, there is still insufficient data to allow the formation of strict guidelines for the management of bifurcation culprit lesions in both STEMI and non-STEMI, as well as to decide whether such guidelines are needed or possible. Therefore, the study aim is to analyze the long-term outcomes after PCI for coronary bifurcation in AMI in the real-world population of patients and assess the influence of clinical diagnosis on the outcomes after PCI.

## 2. Material and Methods

### 2.1. Study Design

The all-comer Zabrze–Opole Registry enrolled patients with coronary bifurcation lesion treated with PCI and implantation of DES. The enrollment was conducted in two tertiary cardiac centers (2nd Department of Cardiology, Zabrze and Department of Cardiology, University Hospital, Opole, Poland) from January, 1st 2011 to December, 31st 2014. Registry exclusion criteria were as follows: non-bifurcation lesion on coronary angiogram, treatment of more than one lesion in the same patient, implantation of BMS and lack or insufficient data required for the registry. For the current analysis, patients were stratified according to clinical diagnosis into AMI and no-AMI groups. AMI was defined according to the current guidelines as non-ST-elevation myocardial infarction (NSTEMI) or ST-elevation myocardial infarction (STEMI) [2,3,4]. By design, basic clinical, angiographic and procedural characteristics were retrospectively collected from medical records. Angiographic and procedural characteristics of bifurcation lesions recorded for the registry were: diameter, stenosis severity and lesion length of the main branch (MB) and side branch (SB); Medina classification [5]; bifurcation angle; initial and final Thrombolysis in myocardial infarction (TIMI) flow; stenting technique; the use of proximal optimization technique (POT); initial and final kissing balloons; predilatation and postdilatation inflation pressure; maximal inflation pressure in MB and SB; contrast volume; procedure duration; radiation time and dose. Thrombus was diagnosed when macroscopically visible from coronary angiogram and/or reported in the medical record by treating physician and/or when a thrombectomy had been attempted during the procedure. True bifurcation was defined as Medina 1-1-1, 0-1-1, or 1-0-1.

### 2.2. Follow-Up

The minimal duration of the follow-up was one year. All information was obtained from medical records of the enrolling centers. If no information was available, phone contact was attempted. In case of phone contact failure, information on clinical endpoints was obtained from the National Health Care System.

Major adverse cardiac events were considered as related to target lesion failure (MACE-TLF) and were defined by TLF-related death, TLF-related non-fatal myocardial infarction and target lesion revascularization (TLR). Death related to TFL was defined as death that occurred after coronary bifurcation stenting procedure in which the role of the device was clear and was followed by AMI or new HF, leading, finally, to death or by death directly (adapted from Cultip et al. [6]). Stent thrombosis (ST) was the rate of definite or possible ST defined as acute, subacute, late and cumulative, according to the definitions of endpoints for clinical trials [6].

### 2.3. Statistics

Variables were checked for normality of distribution with the Shapiro–Wilks test. Continuous variables are presented as mean ± SD or median (25th and 75th percentile) and were compared with the Student-t test or the Mann–Whitney test. Categorical variables are presented as percentages and were compared with the chi^2^ test or the Fisher’s exact test when appropriate. The parameters were tested with a univariate Cox proportional hazard model for significant independent influence on endpoint. The propensity score of the probability of AMI was determined with a logistic regression model and included in the hazard model analysis. A Kaplan–Meier analysis for MACE and its components was performed, adjudicated for chosen variables either clinically relevant or significant in the univariate analysis. All tests were two-tailed and values of *p* < 0.05 was considered significant. All analyses were performed with SPSS version 22.0 (SPSS Inc., Chicago, IL, USA)

## 3. Results

### 3.1. Baseline

During the enrollment period, 3550 PCIs were performed. Of them, 1300 were excluded due to the implantation of BMS, 646 PCIs were excluded due to more than one lesion treated in the same patient and 76 further PCIs were excluded due to lack of, or insufficient data required for the registry. From the remaining 1528 PCIs, 306 cases were PCI for a bifurcation lesion and were included into the registry. Of them, 113 were diagnosed with AMI (31 STEMI and 82 NSTEMI). The clinical profile of the patients with AMI showed a higher incidence of hypertension and lower but preserved or medium reduced ejection fraction. More patients in the non-AMI group had a history of prior AMI and prior PCI (Table 1).

The baseline angiographic profile was similar between groups, with higher incidence of thrombus in the AMI group (Table 2).

A comparison of procedural characteristics showed that PCI in the AMI group was conducted more frequently with predilatation, with 2^nd^ generation DES and higher maximal inflation pressure. For the PCI procedure in AMI, the predominantly involved artery was LAD (76%), the most common technique was provisional T-stenting (77%), SB protection was used in 68% of patients, SB stenting in 27%, final kissing balloons in 21% and POT in 24% of patients.

Regarding postprocedural success rates, TIMI flow grade 3 was achieved with similar success in the AMI and non-AMI groups in MB (98% vs. 97%, respectively; *p* = 0.48) and SB (91% vs. 94%, respectively; *p* = 0.41). Any decrease in TIMI flow was observed in 3.5% and 3.1% of patients, respectively; *p* = 0.84. The rate of residual stenosis in MB was small and equal in both groups (1.8 vs. 0.5%; *p* = 0.28), also equal in SB (19% vs. 19%; *p* = 0.99) (Table 2). SB occlusion occurred in 0.9% vs 0.5% of patients; *p* = 1.0.

There was no statistically significant difference between groups in antithrombotic regiment with 99% vs. 100% of DAPT in AMI vs. non-AMI groups (*p* = 0.70) and 9.7% vs. 9.3% for TAP, respectively (*p* = 0.91).

The side branch was stented in 91 cases (30%). Provisional T-stenting was used in 17 (23%), Crush in 21 (23%), V-stenting in 4 (4.4%) and T-stenting in 44 (48%) cases of SB stenting. A dedicated stent was implanted in 16 (18%) patients. The initial kissing balloons technique was used in 11 (12%) cases and the procedure was finalized with kissing balloons in 42 (46%) cases.

### 3.2. Clinical Outcome

Clinical follow-up was obtained in all patients. All patients completed a minimum of 1 year follow-up and the median follow-up for the study population was 3.6 years. At that time, the MACE-TLF rate was 12% in the AMI group and 15% in the non-AMI group (*p* = 0.46) with equal rates of TLR, AMI-TLF and death-TLF (Table 3). There was no difference in cumulative ST (2.7% vs. 0.5%, respectively; *p* = 0.14), with no difference in acute ST (0% vs. 0.5%, respectively; *p* = 1.0), no cases of subacute ST and significant difference in late ST (2.7% vs 0%, respectively; *p* = 0.02) (Table 3).

An additional analysis between STEMI and non-STEMI groups was performed. There was no difference in baseline characteristics between groups; statistically significant differences in angiographic and procedural characteristics were found for PCI for LAD, thrombus, stenosis severity (which were more common in the STEMI group (*p* = 0.007, <0.001 and 0.001, respectively)) and number of DES per lesion, initial TIMI flow in MB and SB, SB diameter and contract volume, which were higher in the non-STEMI group (*p* = 0.04, <0.001, <0.001, 0.025 and 0.001, respectively). No differences were found in the incidence of MACE and ST (Supplementary Tables S1–S3).

### 3.3. Predictors of Clinical Outcome

The diagnosis of AMI was not a significant risk factor of MACE-TLF in the general population in the univariate analysis (HR, 0.19; 95% CI, 0.03–1.4; *p* = 0.10) or in the model adjusted with propensity score (HR, 0.76; 95% CI, 0.39–1.49; *p* = 0.43). No significance was reached for ST in univariate (HR, 5.07; 95% CI, 0.53–48.72; *p* = 0.16) and multivariate adjusted analysis (HR, 5.84; 95% CI, 0.13–265.3; *p* = 0.37) either.

The detailed results of the univariate Cox regression model for prediction of MACE-TLF in AMI and non-AMI groups are depicted in Table 4. In the AMI group, factors that reached statistical significance were: PCI in LAD (hazard ratio (HR), 0.33; 95% confidence interval (CI) 0.11–0.98; *p* = 0.045), PCI in RCA (HR, 6.19; 95% CI, 1.33–28.87; *p* = 0.02), SB diameter >3 mm (HR, 12.92; 95% CI, 1.53–108.9; *p* = 0.019) and SB maximal inflation pressure (HR, 2.25; 95% CI, 1.16–4.35; *p* = 0.017). In the non-AMI group, significant predictors of MACE-TLF were maximal inflation pressure (HR, 1.03; 95% CI, 1.002–1.06; *p* = 0.04), SB diameter >3 mm (HR, 4.14; 95% CI, 1.44–11.95; *p* = 0.009), V-stenting (HR, 5.41; 95% CI, 1.28–22.91; *p* = 0.02), predilatation pressure (HR, 0.74; 95% CI, 0.58–0.96; *p* = 0.02), procedure time (HR, 1.01; 95% CI, 1.001–1.03; *p* = 0.03), radiation time (HR, 1.03; 95% CI, 1.003–1.07; *p* = 0.03) and radiation dose (HR, 1.0; 95% CI, 1.0–1.001; *p* = 0.03).

**Figure 1:**
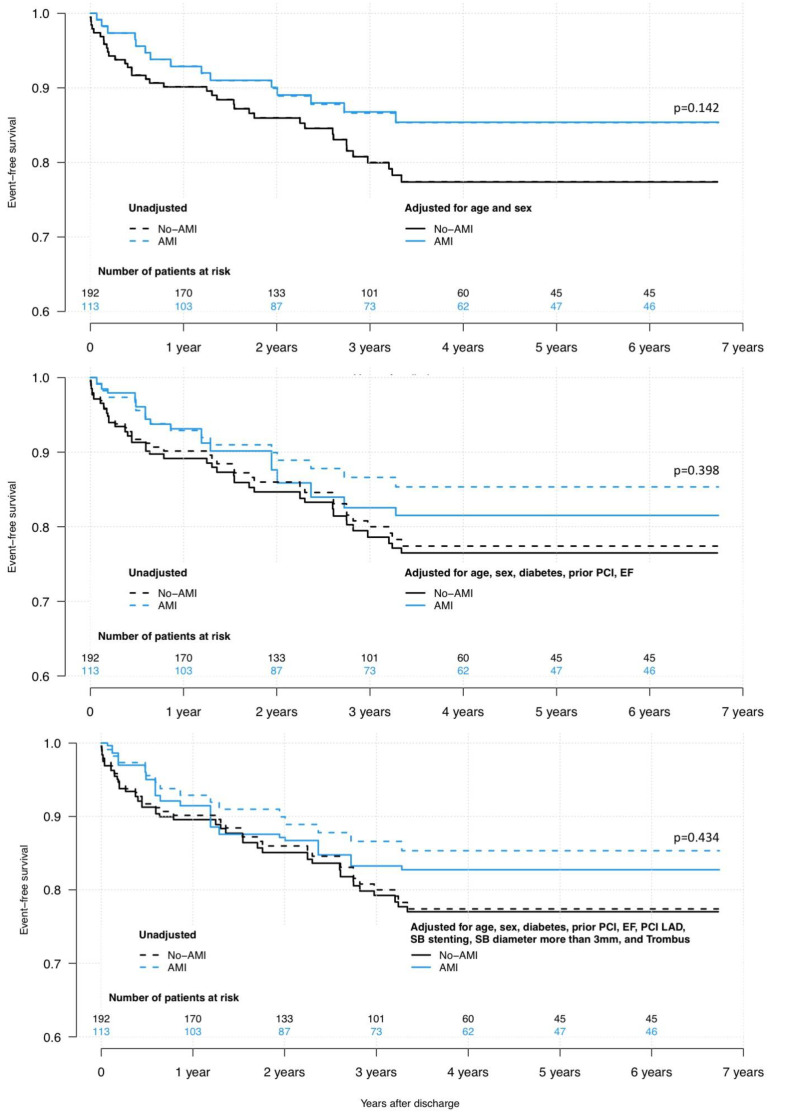
Kaplan–Meier analysis of MACE-TLF free survival. AMI, acute myocardial infarction; PCI, percutaneous coronary intervention; EF, ejection fraction; LAD, left anterior descending artery; SB, side branch.

Three models of Kaplan-Meier analysis between AMI and non-AMI groups for MACE-TLF were performed: 1, adjudicated for age and sex; 2, adjudicated for age, sex, diabetes, prior PCI and EF; 3, adjudicated with previous variables and PCI LAD, SB stenting, SB > 3 mm and thrombus. None of the analysis showed statistically significant difference in event-free survival between AMI and non-AMI groups (Figure 1).

An additional analysis was performed for TLF as the endpoint in the Cox hazard model.

In the AMI group, independent predictors of TLF in the univariate analysis were PCI in LAD (HR, 0.23; 95% CI, 0.06–0.87; *p* = 0.03), PCI in RCA (HR, 9.08; 95% CI, 1.81–45.43; *p* = 0.007), direct stenting (HR, 6.31; 95% CI, 1.58–25.26; *p* = 0.009) and SB diameter >3 mm (HR, 17.46; 95% CI, 1.92–158.9; *p* = 0.011). In the non-AMI group, none of the studied parameters reached statistical significance in the univariate Cox model. The analogic Kaplan–Meier analysis for the three risk models was performed and revealed no statistically significant difference between AMI and non-AMI groups for TLF-free survival (model 1, *p* = 0.72; model 2, *p* = 0.97; model 3, *p* = 0.96; Figure S1A–C), nor for survival (model 1, *p* = 0.34; model 2, *p* = 0.34; model 3, *p* = 0.35; Figure S1D–F).

Regarding the safety profile, the Cox regression model indicated only maximal inflation pressure in MB as an independent predictor of ST (HR, 0.81; 95% CI, 0.65–0.998; *p* = 0.048).

## 4. Discussion

The Zabrze–Opole registry is a real-life, ongoing, all-comer, two-center registry of patients with coronary bifurcation treated with DES. The current analysis, based on a pilot population, focuses on outcomes after PCI in coronary bifurcation as the culprit lesion in AMI and shows the following findings: First, the diagnosis of AMI was not associated with increased risk of MACE-TLF or ST after PCI in bifurcation culprit lesion. Postprocedural success rates were similar in AMI and non-AMI groups. Second, the risk of MACE-TLF was dependent on single variables (the treated lesion in the AMI but not in the non-AMI group and was the highest for non-LAD lesions, especially RCA). Considering the baseline angiographic profile of the patients with AMI, the SB diameter and culprit vessel, were independent risk factors of MACE-TLF and TLR. Third, in the cluster of clinical and angiographic variables, the significance of predictive value of single variables no longer persisted, which leads to the conclusion that the risk of PCI for bifurcation lesion is high irrespective of clinical condition (AMI/non-AMI), taking into account the complex risk profile of the patients. Fourth, the PCI of coronary bifurcation in AMI is saddled with a higher rate of late ST; however, only the inflation pressure in MB was a significant risk factor of ST.

The presented analysis adds some insights to the current discussion on optimal strategy for the management of bifurcation culprit lesions in AMI. Despite extensive evidence in this field supported by the rapid development of techniques and technology, the status on strict recommendations is still inconclusive. Previous reports showed that the PCI of bifurcation culprit lesion in AMI, despite higher complexity and increased risk of procedural and clinical complications associated with PCI for bifurcation, carried similar clinical risk to single-vessel culprit lesions [7,8].

Our study is oriented at analyzing if an acute clinical setting has any influence on the procedure of bifurcation stenting itself or subsequent clinical outcome and on indicating possible factors for improving prognosis. Giving comparable PCI strategies used in AMI and non-AMI patients, the diagnosis of AMI did not impair the clinical outcome, nor did it increase the rates of measures of procedural failure. However, MACE-TLF was related to both angiographic status and procedural characteristics—different for AMI and non-AMI patients. Of notice, a relatively small fraction of patients was reported as having thrombus in the AMI group. It was mainly due to the retrospective character of the registry and the lack of routinely used intravascular visualization techniques. This might bias the prognostic outcome and is probably one of the factors related to the equal rate of MACE irrespectively of the initial diagnosis of AMI. On the other hand, previous studies on coronary bifurcation stenting also reported that an acute clinical setting was not a risk factor for MACE [9]. In other words, different parameters may influence the clinical course after PCI for bifurcation lesions in AMI and non-AMI patients, but the high complexity of the procedure itself results in equally high long-term risk, irrespective of clinical condition and complex risk profile of patients.

### 4.1. Anatomy

Bifurcation culprit lesions are common in AMI, constitute one-fourth of primary PCI and are most commonly located in LAD [7,8]. However, we found that it is PCI in the non-LAD bifurcation, especially located in RCA, which is at higher risk of adverse events. Such result might be inconsistent with numerous previous reports on higher risk after PCI in LAD [10,11] or LM [12].

Considering the special character of bifurcation lesions, with a higher risk of potential acute occlusion of SB and in-stent restenosis [13,14], the reason for a worse outcome after PCI within RCA might be attributable to coronary anatomy with a relatively smaller cross-sectional area and a higher angle of the right-ventricular branch leading to hemodynamic conditions more prone to atherosclerosis formation and restenosis [15].

The second factor that should be considered while estimating prognosis in PCI for bifurcation culprit lesions is the diameter of the SB. A diameter >3 mm was a factor of MACE-TLF and was also predictive of TLR in the long term in the AMI group. A large SB diameter, contrary to the diameter of the MB, is a known risk factor and one of the variables defining complex bifurcation lesions [16]. In patients without AMI, the outcome was not dependent on the treated vessel, nor on the SB diameter.

### 4.2. Procedure

One of the therapeutic approaches specific for bifurcation lesions is the need for choosing the treatment strategy before the procedure, that is consistently recommended by experts’ opinions [17], largely limited by the setting of AMI. Acute conditions require timely adaptation to the angiographic status of the patients. Our analysis brings up parameters of potential utility in such case. From all analyzed procedural variables only direct stenting and SB inflation pressure were independent risk factors for TLR and MACE-TLF, respectively. There was no influence on the rate of MACE-TLF by stenting technique, provisional-T-stenting, the most common in the study group, nor 2-stent strategy. In light of these findings, optimal preparation before stenting is of utmost importance, also when treating bifurcation culprit lesions in AMI. Following the recommendations, provisional stenting should be the method of choice in the majority of cases for the restoration of the blood flow [17], as one-stent techniques are predominant over two-stent techniques [18,19]. According to some reports, elective SB involvement might be advantageous, especially in vulnerable plaques in ACS patients [20]. In case the SB is involved, attention should be paid to the inflation pressure.

### 4.3. Stent Thrombosis and MACE

The optimization of the procedure with proper stent sizing and deployment is important, especially in patients with acute coronary syndrome and high thrombotic burden [21]. In the studied population, POT and final kissing balloons were not performed in all patients. Nevertheless, the rates of cumulative ST were very low. Contrary to previously published data [22], the rate of cumulative ST was driven mainly by late events. It is not surprising, considering that the major concern accompanying the implantation of DES is very late ST [23,24]. The in-stent thrombosis in DES is a derivative of several factors [25]. Dealing with a priori high-risk culprit lesion specific for AMI, the single significant risk factor for ST in the study population was the inflation pressure in the MB, with higher risk with lower pressure. Again this confirms that optimal stent deployment is crucial in PCI for bifurcation culprit lesion in AMI.

All the above-mentioned factors are of potential influence on the clinical outcome but the significance of predictive value of single variables no longer persisted in complex risk modeling. Due to limitations of the statistical analyses, the reliable methodology needed to understand the risk of MACE in the cluster of clinical and angiographic variables beyond clinical diagnosis is not defined and the results should be considered an approximation.

### 4.4. Limitations

The nature of a registry is retrospective, which limits the acquisition of more specific angiographic or procedural parameters in a routine way. The relatively small study sample limited a subgroup analysis, for example, a more detailed view of patients with large SB or SB stenting and limited possibilities of reducing bias by propensity score matching.

The occasional use of intravascular imaging techniques in patients enrolled in the registry was a factor depreciating exact evaluation of direct outcomes of PCI. Parameters such as SB occlusion after PCI in the MB or strut apposition, plaque characteristics, the minimum lumen area and carina shift would shed light and possibly uncover differences between PCI for bifurcation in AMI and non-AMI patients beyond clinical outcomes [26].

The analysis may not fully allow to extrapolate the outcomes for complex stenting techniques for bifurcation lesions, as, in the majority of cases, provisional T-stenting was the applied stenting technique and, in only one-third of the patients, side-branch stenting was applied, which was secondary to the angiographic profile of the studied population and related to a relatively high rate of non-true bifurcation lesions.

Further, large up-to-date studies are needed to establish the optimal strategy for coronary bifurcation treatment and identify potential risk factors of adverse short- and long-term events.

## 5. Conclusions

In the all-comer registry of coronary bifurcations treated with DES, AMI was not a risk factor for MACE-TLF or ST. Nevertheless, to reduce the rate of MACE-TLF, PCI for bifurcation culprit lesions in AMI should be performed carefully in case of culprit lesions located in the RCA and in case of high SB diameter. Side-branch stenting should be performed paying attention to high inflation pressure, as it remained the only risk factor of poor prognosis. On the contrary, the lower the inflation pressure in the MB, the higher the risk for ST.

## Figures and Tables

**Table 1:** Clinical characteristics.

Characteristic	AMI (*n* = 113)	Non-AMI (*n* = 193)	*p*-Value
Male sex	77 (69)	130 (67)	0.89
Age (years)	64 (58–74)	66 (60–73)	0.49
BMI (kg/m^2^)	27.2 (25.4–29.7)	28.4 (25.6–31.2)	0.19
Obesity	23 (20)	59 (31)	0.052
Renal insufficiency	12 (11)	14 (7.3)	0.31
Ejection fraction (%)	45 (40–55)	55 (45–60)	0.001
Diabetes mellitus	35 (31)	67 (35)	0.50
Hypertension	92 (81)	174 (90)	0.029
Dyslipidemia	88 (78)	151 (78)	0.94
Smoker	45 (40)	82 (43)	0.65
Familial history of CAD	31 (27)	54 (28)	0.92
Prior AMI	21 (19)	64 (33)	0.006
Prior PCI	18 (16)	81 (42)	<0.001
Prior CABG	8 (7.2)	20 (10)	0.34
Carotid atherosclerosis	5 (4.4)	3 (1.6)	0.13
PAD	11 (9.7)	14 (7.3)	0.45

Data are presented as *n* (%) or median (25th–75th percentile). AMI, acute myocardial infarction; BMI, body mass index; CAD, coronary artery disease; PCI, percutaneous coronary intervention; CABG, coronary artery bypass grafting; PAD, peripheral artery disease.

**Table 2:** Angiographic and procedural characteristics.

Characteristic	AMI (*n* = 113)	Non-AMI (*n* = 193)	*p*-Value
**Femoral approach**	102 (90)	162 (84)	0.12
**Culprit vessel**			
LM	17 (15)	21 (11)	0.29
LAD	86 (76)	133 (69)	0.18
Cx	18 (16)	48 (25)	0.07
RCA	6 (5.3)	5 (2.6)	0.22
**Thrombus**	19 (17)	1 (0.5)	<0.001
**Ostial lesion**	39 (35)	52 (27)	0.16
**Restenosis**	3 (2.7)	12 (6.2)	0.27
**Calcifications**	28 (18)	27 (14)	0.43
**DES 2nd generation**	73 (65)	100 (51)	0.03
**No of DES/lesion**	1 (1;2)	1 (1;2)	0.15
**Length of DES/lesion (mm)**	25 (18–33)	26 (18–33)	0.73
**Direct stenting**	29 (26)	90 (47)	<0.001
**Maximal inflation pressure (atm)**	16 (12–18)	14 (12–16)	0.01
**GP IIbIIIa**	19 (17)	4 (2.1)	<0.001
**Dedicated stent**	13 (12)	31 (16)	0.27
**True bifurcation**	62 (55)	113 (59)	0.53
**Main branch**			
Stenosis severity	90 (80–99)	80 (70–90)	<0.001
Diameter	3.0 (2.75–3.25)	3.0 (2.75–3.25)	0.58
diameter <2.5 mm	6 (5.3)	9 (4.7)	0.80
diameter <2.75 mm	27 (24)	39 (20)	0.45
diameter <3 mm	45 (40)	74 (38)	0.80
diameter <3.25 mm	80 (71)	133 (69)	0.73
diameter <3.5 mm	88 (78)	147 (76)	0.73
diameter <3.75 mm	106 (94)	176 (91)	0.41
diameter <4 mm	107 (95)	181 (94)	0.75
Initial TIMI flow	3 (2–3)	3 (3–3)	<0.001
Final TIMI flow	3 (3–3)	3 (3–3)	0.48
MB residual stenosis >10%	2 (1.8)	1 (0.5)	0.28
Stent length (mm)	23 (18–28)	23 (18–30)	0.61
Max inflation pressure (atm)	14 (12–16)	14 (12–16)	0.16
**Side branch**			
Stenosis severity	55 (0–90)	60 (0–80)	0.47
Diameter	2.25 (2.0–2.5)	2.25 (2.0–2.75)	0.053
diameter > 2 mm	63 (56)	125 (65)	0.12
diameter >2.25	43 (38)	93 (48)	0.09
diameter >2.5	19 (17)	57 (30)	0.013
diameter >2.75	15 (13)	34 (18)	0.32
diameter >3	2 (1.8)	8 (4.1)	0.26
Initial TIMI flow	3 (2–3)	3 (3–3)	<0.001
Final TIMI flow	3 (3–3)	3 (3–3)	0.41
SB residual stenosis >10%	21 (19)	36 (19)	0.99
Stent length	15 (12–22)	14.5 (12–18)	0.34
Max inflation pressure	12 (12–14)	14 (12–14)	0.99
**SB occlusion**	1 (0.9)	1 (0.5)	1.0
**Bifurcation angle <90%**	81 (72)	119 (62)	0.08
**Stenting technique**			
Provisional T-stenting	87 (77)	139 (72)	0.29
Crush	6 (5.3)	15 (7.8)	0.41
V-stenting	0 (0)	4 (2.1)	0.30
T-stenting	18 (16)	32 (17)	0.88
**Crossover**	15 (13)	33 (17)	0.38
**GW-SB**	77 (68)	126 (65)	0.61
**POT**	27 (24)	41 (21)	0.58
**Predilatation pressure**	12 (10–14)	12 (10–14)	0.75
**Initial Kissing Balloons**	5 (4.4)	10 (5.2)	0.77
**Final Kissing Balloons**	24 (21)	50 (26)	0.36
**SB stenting**	31 (27)	60 (31)	0.50
**Procedure time (min)**	65 (50–80)	60 (50–80)	0.75
**Contrast volume (ml)**	150 (120–170)	150 (100–200)	0.91
**Radiation time (min)**	15.5 (9.5–20)	14.5 (10–23)	0.93
**Radiation dose (mGy)**	1343 (902–1976)	1128 (853–1655)	0.15

Data are presented as *n* (%) or median (25th–75th percentile). AMI, acute myocardial infarction; LM, left main; LAD, left anterior descending artery; Cx, circumflex artery; RCA, right coronary artery; DES, drug eluting stent.

**Table 3:** Clinical outcomes.

Characteristic	AMI (*n* = 113)	Non-AMI (*n* = 193)	*p*-Value
**Stent Thrombosis**			
Acute	0 (0)	1 (0.5)	1
Subacute	0 (0)	0 (0)	-
Late	3 (2.7)	0 (0)	0.02
Cumulative	3 (2.7)	1 (0.5)	0.14
**MACE**			
MACE-TLF	13 (12)	28 (15)	0.46
Death-TLF	2 (1.8)	7 (3.6)	0.49
AMI-TLF	7 (6.2)	15 (7.8)	0.61
AMI-TLF + TLR	5	11	0.23
TLR	9 (8.0)	17 (8.8)	0.80

Data are presented as *n* (%). AMI, acute myocardial infarction; MACE, major adverse cardiac events; TLF, target lesion failure; TLR, target lesion revascularization.

**Table 4:** Univariate Cox proportional hazard model for risk prediction of MACE-TLF.

Characteristic	*p*-Value	HR	95% CI	*p*-Value	HR	95% CI
	AMI			Non-AMI		
**Clinical** parameters *						
EF	0.78	1.01	0.96–1.05	0.74	1.02	0.92–1.12
Prior AMI	0.70	1.29	0.35–4.68	0.94	0.91	0.08–10.09
Prior PCI	0.17	2.3	0.71–7.48	0.80	1.36	0.12–15.0
Hypertension	0.85	1.16	0.26–5.23	0.63	26.6	0-
**Hemodynamic parameters**						
PCI LM	0.09	2.81	0.87–9.16	0.83	0.87	0.26–2.90
PCI LAD	0.045	0.33	0.11–0.98	0.98	0.99	0.45–2.19
PCI Cx	0.14	2.41	0.74–7.84	0.70	1.17	0.52–2.67
PCI RCA	0.02	6.19	1.33–28.87	0.59	0.05	0–3404
Thrombus	—		—	0.81	0.05	-
Ostial lesion	0.87	0.91	0.29–2.82	0.74	0.32	0.32–1.73
Restenosis	0.25	3.29	0.43–25.37	0.65	0.63	0.09–4.62
No of DES/lesion	0.38	0.52	0.12–2.27	0.57	1.19	0.66–2.13
Length of DES/lesion	0.52	0.98	0.92–1.04	0.38	1.01	0.99–1.04
Direct stenting	0.07	2.71	0.91–8.06	0.84	0.93	0.44–1.95
Max inflation pressure	0.97	0.996	0.84–1.18	0.04	1.03	1.002–1.06
GP IIbIIIa	0.91	0.92	0.2–4.13	0.59	0.05	0–2975
Dedicated stent	0.85	0.82	0.11–6.35	0.14	0.34	0.08–1.43
True bifurcation	0.87	0.91	0.31–2.71	0.14	1.87	0.82–4.24
**Main branch**						
Stenosis severity	0.28	0.99	0.96–1.01	0.80	1.0	0.97–1.03
Diameter	0.76	1.2	0.37–3.96	0.33	1.44	0.70–2.98
diameter <2.5 mm	0.55	0.05	0–1114	0.73	0.70	0.10–5.15
diameter <2.75 mm	0.44	0.55	0.12–2.49	0.36	0.61	0.21–1.76
diameter <3 mm	0.94	0.96	0.31–2.92	0.41	0.72	0.32–1.59
diameter <3.25 mm	0.43	0.64	0.21–1.95	0.36	0.70	0.33–1.50
diameter <3.5 mm	0.97	0.97	0.27–3.53	0.31	0.66	0.30–1.46
diameter <3.75 mm	0.54	22.2	0.001–416138	0.34	0.60	0.21–1.71
diameter <4 mm	0.57	21.92	0.001–798210	0.95	0.95	0.23–4.02
Initial TIMI flow	0.83	0.99	0.91–1.08	0.95	0.98	0.46–2.08
Final TIMI flow	0.75	4.37	0–42192	0.55	0.80	0.37–1.70
Stent length	0.71	1.01	0.94–1.09	0.37	1.01	0.98–1.05
Max inflation pressure	0.76	0.98	0.88–1.10	0.49	0.95	0.83–1.09
**Side branch**						
Stenosis severity	0.60	0,996	0.98–1.01	0.52	1.0	0.99–1.02
Diameter	0.59	1.42	0.4–5.06	0.26	1.55	0.73–3.27
diameter > 2 mm	0.91	0.94	0.32–2.8	0.20	1.76	0.75–4.15
diameter >2.25	0.51	1.44	0.49–4.3	0.50	1.29	0.61–2.72
diameter >2.5	0.43	1.68	0.46–6.13	0.64	1.21	0.55–2.67
diameter >2.75	0.23	2.22	0.61–8.09	0.55	1.32	0.53–3.25
diameter >3	0.019	12.92	1.53–108.9	0.009	4.14	1.44–11.95
Initial TIMI flow	0.23	1.65	0.72–3.78	0.60	1.20	0.61–2.38
Final TIMI flow	0.91	1.06	0.43–2.62	0.73	0.91	0.52–1.59
Stent length	0.83	0.98	0.85–1.14	0.55	0.97	0.87–1.08
Max inflation pressure	0.017	2.25	1.16–4.35	0.17	1.26	0.90–1.75
Bifurcation angle <90%	0.34	0.48	0.11–2.16	0.96	1.02	0.50–2.09
**Stenting technique**						
Provisional T-stenting	0.23	3.51	0.46–26.99	0.54	0.78	0.35–1.71
Crush	0.58	0.05	0–2659	0.93	1.07	0.25–4.53
V-stenting	—	—	—	0.02	5.41	1.28–22.91
T-stenting	0.43	0.44	0.06–3.4	0.76	0.85	0.29–2.44
Crossover	0.65	1.41	0.31–6.41	0.82	0.88	0.31–2.55
GW-SB	0.48	1.60	0.44–5.82	0.80	0.91	0.42–1.94
POT	0.59	1.38	0.43–4.49	0.72	1.17	0.50–2.78
Predilatation pressure	0.77	1.05	0.77–1.44	0.02	0.74	0.58–0.96
Initial Kissing Balloons	0.43	2.28	0.29–17.73	0.09	2.84	0.85–9.48
Final Kissing Balloons	0.25	0.30	0.04–2.3	0.38	0.65	0.25–1.71
Contrast volume	0.49	1,0	0.99–1.01	0.07	1.01	1.0–1.01
Procedure time	0.32	1.01	0.99–1.03	0.03	1.01	1.001–1.03
Radiation time	0.37	1.03	0.97–1.09	0.03	1.03	1.003–1.07
Radiation dose	0.22	1	1–1.001	0.03	1.0	1–1.001
DES 2nd generation	0.051	0.31	0.1–1.004	0.11	0.54	0.25–1.16
SB stenting	0.65	0.70	0.16–3.18	0.58	0.78	0.33–1.85

* Clinical parameters significantly different between AMI and non-AMI groups. MACE-TLF, major adverse cardiac event related to target lesion failure; AMI, acute myocardial infarction; HR, hazard ratio; CI, confidence interval; EF, ejection fraction; PCI, percutaneous coronary intervention; LM, left maim; LAD, left anterior descending artery; Cx, circumflex artery; RCA, right coronary artery; DES, drug eluting stent; MB, main branch; SB, side branch; TIMI, thrombosis in myocardial infarction; GW, guide wire; POT, provisional optimization technique.

## Data Availability

The data presented in this study are available on request from the corresponding author. The data are not publicly available due to procedural and Institution Data Policy reasons.

## Supplementary Materials

The following are available online at https://www.mdpi.com/article/10.3390/jcdd8110155/s1, Figure S1: Kaplan–Meier analysis of TLR-free survival (A–C) and survival (D–F) for STEMI and non-STEMI; Table S1: Clinical characteristics in STEMI vs. non-STEMI, Table S2: Angiographic and procedural characteristics in STEMI vs. non-STEMI; Table S3: Clinical outcomes in STEMI vs. non-STEMI.
