# Supplementary material for: Real-Life Outcomes of Coronary Bifurcation Stenting in Acute Myocardial Infarction (Zabrze–Opole Registry)
Source: J Cardiovasc Dev Dis. 2021 Nov 11;8(11):155. doi: 10.3390/jcdd8110155 (PMC8619945; doi:10.3390/jcdd8110155)
Supplement: Supplementary file 1 [file jcdd-08-00155-s001.zip › Tables_STEMIvsNSTEMI.pdf]

Table S1. Clinical characteristics.

Characteristic	STEMI (n=31)	NonSTEMI (n=82)	P value
**Male sex**	19 (61)	58 (71)	0.34
**Age (years)**	63 (58-74)	64 (59-74)	0.63
**BMI (kg/m^2)**	27 (25-30)	28 (26-30)	0.60
**Obesity**	4 (13)	29 (23)	0.23
**Renal insufficiency**	3 (9.7)	9 (11)	0.84
**Ejection fraction (%)**	44 (35-50)	49 (40-60)	0.08
**Diabetes mellitus**	10 (32)	25 (31)	0.86
**Hypertension**	24 (77)	68 (83)	0.50
**Dyslipidemia**	26 (84)	62 (76)	0.35
**Smoker**	11 (36)	34 (42)	0.56
**Familial history of CAD**	5 (16)	26 (32)	0.10
**Prior AMI**	4 (13)	17 (21)	0.34
**Prior PCI**	4 (13)	14 (17)	0.59
**Prior CABG**	0 (0)	8 (9.8)	0.07
**Carotid atherosclerosis**	0 (0)	5 (6.1)	0.32
**PAD**	2 (6.5)	9 (11)	0.72

Data are presented as n (%) or median (25th-75th percentile).

AMI – acute myocardial infarction; BMI – body mass index; CAD – coronary artery disease; PCI – percutaneous coronary intervention; CABG – coronary artery bypass grafting; PAD – peripheral artery disease

Outcomes should be interpreted with caution to small group sizes.

Table S2. Angiographic and procedural characteristics

**Characteristic**	**STEMI (n=31)**	**NonSTEMI (n=82)**	**P value**
**Femoral approach**	30 (97)	72 (88)	0.28
**Culprit vessel**			
LM	4 (13)	13 (16)	0.77
LAD	29 (94)	57 (70)	**0.007**
Cx	2 (6.5)	16 (20)	0.15
RCA	1 (3.2)	5 (6.1)	1.0
**Thrombus**	11 (36)	8 (9.8)	**<0.01**
**Ostial lesion**	14 (45)	25 (31)	0.14
**Restenosis**	0 (0)	3 (3.7)	0.56
**Calcifications**	3 (9.7)	17 (21)	0.27
**DES 2nd generation**	22 (71)	46 (56)	0.17
**No of DES/lesion**	1 (1;1)	1 (1;2)	**0.04**
**Length of DES/lesion (mm)**	23 (18-28)	25 (18-33)	0.22

**Direct stenting**	29 (26)	90 (47)	0.65
**Maximal inflation pressure (atm)**	16 (12-18)	14 (12-16)	0.26
**GP IIbIIIa**	7 (23)	12 (15)	0.31
**Dedicated stent**	4 (13)	9 (11)	0.75
**True bifurcation**	14 (45)	48 (59)	0.20
**Main branch**			
Stenosis severity	99 (90-99)	90 (80-99)	**0.001**
Diameter	3.0 (2.5-3.25)	3.0 (2.75-3.25)	0.77
Initial TIMI flow	2 (1-3)	3 (2-3)	**<0.001**
Final TIMI flow	3 (3-3)	3 (3-3)	0.22
MB residual stenosis >10%	1 (3.2)	1 (1.2)	0.48
Stent length (mm)	23 (18-28)	23 (18-28)	0.59
Max inflation pressure (atm)	16 (12-18)	14 (12-16)	0.28
**Side branch**			
Stenosis severity	40 (0-80)	57.5 (0-90)	0.64
Diameter	2.0 (2.0-2.5)	2.25 (2.0-2.5)	0.07
Diameter >2.5mm	12 (39)	51 (62)	**0.025**

Initial TIMI flow	2 (1-3)	3 (2-3)	**<0.001**
Final TIMI flow	3 (3-3)	3 (3-3)	0.49
SB residual stenosis >10%	2 (6.5)	19 (23)	0.06
Stent length	15 (8-15)	18 (12-22.5)	0.16
Max inflation pressure	12 (10-14)	13 (12-15)	0.14
**SB occlusion**	1 (0.9)	1 (0.5)	1.0
**Bifurcation angle <90%**	21 (68)	60 (73)	0.57
**Stenting technique**			
Provisional T-stenting	25 (81)	62 (76)	0.63
Crush	1 (3.2)	5 (6.1)	1.0
V-stenting	0 (0)	0 (0)	-
T-stenting	4 (13)	14 (17)	0.78
**Crossover**	1 (3.2)	14 (17)	0.06
**GW - SB**	17 (55)	60 (73)	0.06
**POT**	9 (29)	18 (22)	0.45
**Predilatation pressure**	12 (10-14)	12 (10-14)	0.86
**Initial Kissing Balloons**	0 (0)	5 (6.1)	0.32
**Final Kissing Balloons**	3 (9.7)	21 (26)	0.08

**SB stenting**	7 (23)	24 (29)	0.48
**Procedure time (min)**	60 (50-70)	60 (50-80)	0.14
**Contrast volume (ml)**	150 (100-150)	150 (125-190)	**0.001**
**Radiation time (min)**	13 (8-21)	15.5 (10.5-20)	0.79
**Radiation dose (mGy)**	1343 (791-1575)	1355 (910-2019)	0.24

Data are presented as n (%) or median (25th-75th percentile)

AMI – acute myocardial infarction; LM – left main; LAD – left anterior descending artery; Cx- circumflex artery; RCA – right coronary artery; DES – drug eluting stent.

Outcomes should be interpreted with caution to small group sizes.

Table S3. Clinical outcomes.

**Characteristic**	**STEMI (n=31)**	**NonSTEMI (n=82)**	**P value**
**Stent thrombosis**			
Acute	0 (0)	0 (0)	-
Subacute	0 (0)	0 (0)	-
Late	1 (3.2)	2 (2.4)	1.0
Cumulative	1 (3.2)	2 (2.4)	1.0
**MACE-TLF**	1 (3.2)	12 (15)	0.11
Death-TLF	0 (0)	2 (2.4)	1.0
AMI-TLF	1 (3.2)	6 (7.3)	0.67
TLR	1 (3.2)	8 (9.8)	0.44

Data are presented as n (%).

AMI – acute myocardial infarction; MACE – major adverse cardiac events; TLF – target lesion failure; TLR – target lesion revascularization.

Outcomes should be interpreted with caution to small group sizes.
