# Supplementary figures and images for: Real-Life Outcomes of Coronary Bifurcation Stenting in Acute Myocardial Infarction (Zabrze–Opole Registry)
Source: J Cardiovasc Dev Dis. 2021 Nov 11;8(11):155. doi: 10.3390/jcdd8110155 (PMC8619945; doi:10.3390/jcdd8110155)

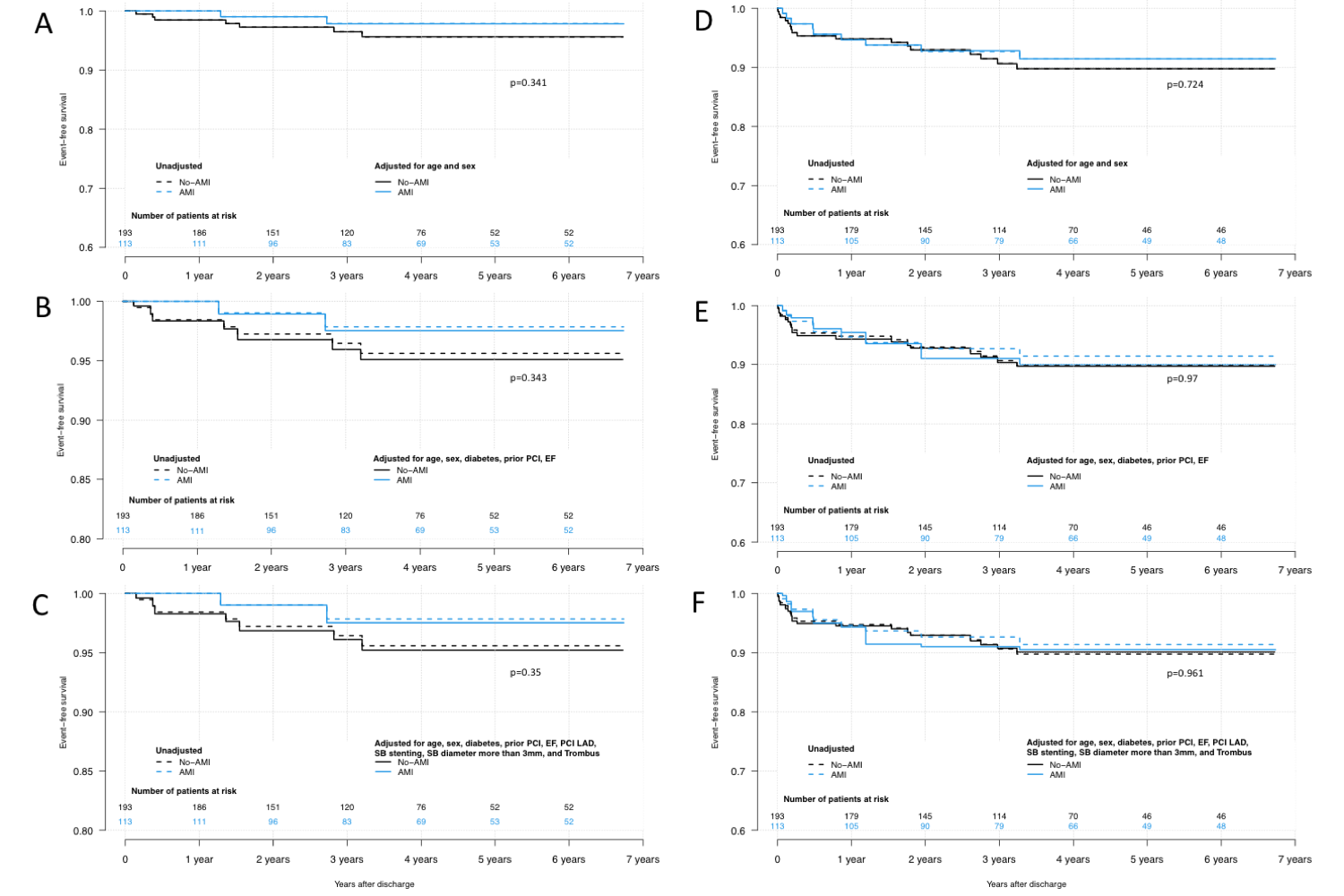

Supplement: Supplementary file 1 [file jcdd-08-00155-s001.zip › Bifurkacje_FigurySuplement2.tiff]
